# Unilateral Purtscher-Like Retinopathy Following Gemcitabine and Bevacizumab Therapy for Recurrent Cervical Carcinoma: A Case Report

**DOI:** 10.7759/cureus.108723

**Published:** 2026-05-12

**Authors:** Madhu Kumar, Rishabh Narula, Pooja Kandula

**Affiliations:** 1 Vitreo-Retina, Sankara Eye Hospital, Guntur, IND

**Keywords:** bevacizumab, chemotherapy-related toxicity, gemcitabine, macular oedema, purtscher-like retinopathy, retina, thrombotic micro-angiopathy

## Abstract

Purtscher-like retinopathy (PLR) is a rare, vision-threatening occlusive microvasculopathy. We report a rare case of unilateral PLR following the initiation of chemotherapy for recurrent cervical carcinoma. A 54-year-old female with recurrent carcinoma of the cervical vault presented with sudden, painless diminution of vision in the left eye (visual acuity: 2/60), five days post-initiation of chemotherapy with gemcitabine and bevacizumab. Fundus examination revealed multiple superficial hemorrhages, cotton wool spots, and Purtscher flecken with macular edema, initially diagnosed elsewhere as a combined central retinal artery and vein occlusion. Systemic laboratory workup was unremarkable. A diagnosis of chemotherapy-induced PLR secondary to localized thrombotic microangiopathy was established. The patient was managed conservatively with close oncological follow-up. Gemcitabine and bevacizumab can precipitate localized thrombotic microangiopathy leading to PLR. Precise funduscopic evaluation is essential to distinguish PLR from combined retinal vascular occlusions to prevent unnecessary interventions and guide appropriate multidisciplinary management.

## Introduction

Purtscher's retinopathy, classically observed with severe trauma, is an exceptionally rare entity, possessing an estimated global incidence of just 0.24 cases per million annually [1]. It is characterized by sudden-onset visual deterioration associated with cotton wool spots, intraretinal hemorrhages, and polygonal areas of inner retinal whitening - the Purtscher flecken - focal infarcts that classically spare the periarteriolar space. Such findings, in the absence of trauma, constitute Purtscher-like retinopathy (PLR). Etiologies include acute pancreatitis or systemic autoimmune diseases.

Drug-induced PLR is exceedingly rare, with only a handful of cases reported in the existing literature [2-4]. Chemotherapeutic agents are well-documented triggers for systemic thrombotic microangiopathy (TMA) [2,3,5]. Furthermore, adding anti-vascular endothelial growth factor (anti-VEGF) agents can exacerbate endothelial dysfunction. We present a case of unilateral PLR that developed shortly after the administration of gemcitabine and bevacizumab for recurrent cervical carcinoma.

## Case presentation

A 54-year-old female presented with complaints of sudden-onset, painless diminution of vision in her left eye for the past two days. Her medical history was significant for carcinoma of the cervix with recurrence in the cervical vault. She had previously undergone five cycles of chemotherapy with carboplatin, paclitaxel, and cyclophosphamide in 2024, and six cycles in 2025 alongside intracavitary brachytherapy, which were all tolerated well. Due to a recent disease recurrence, she had been initiated on a new chemotherapeutic regimen incorporating gemcitabine (1000 mg) and bevacizumab (400 mg). Her visual symptoms commenced five days following the first cycle of this new regimen.

She had no other known systemic comorbidities. She had been evaluated elsewhere and referred to our hospital with a provisional diagnosis of a combined central retinal artery and vein occlusion (CRAO/CRVO).

**Figure 1:**
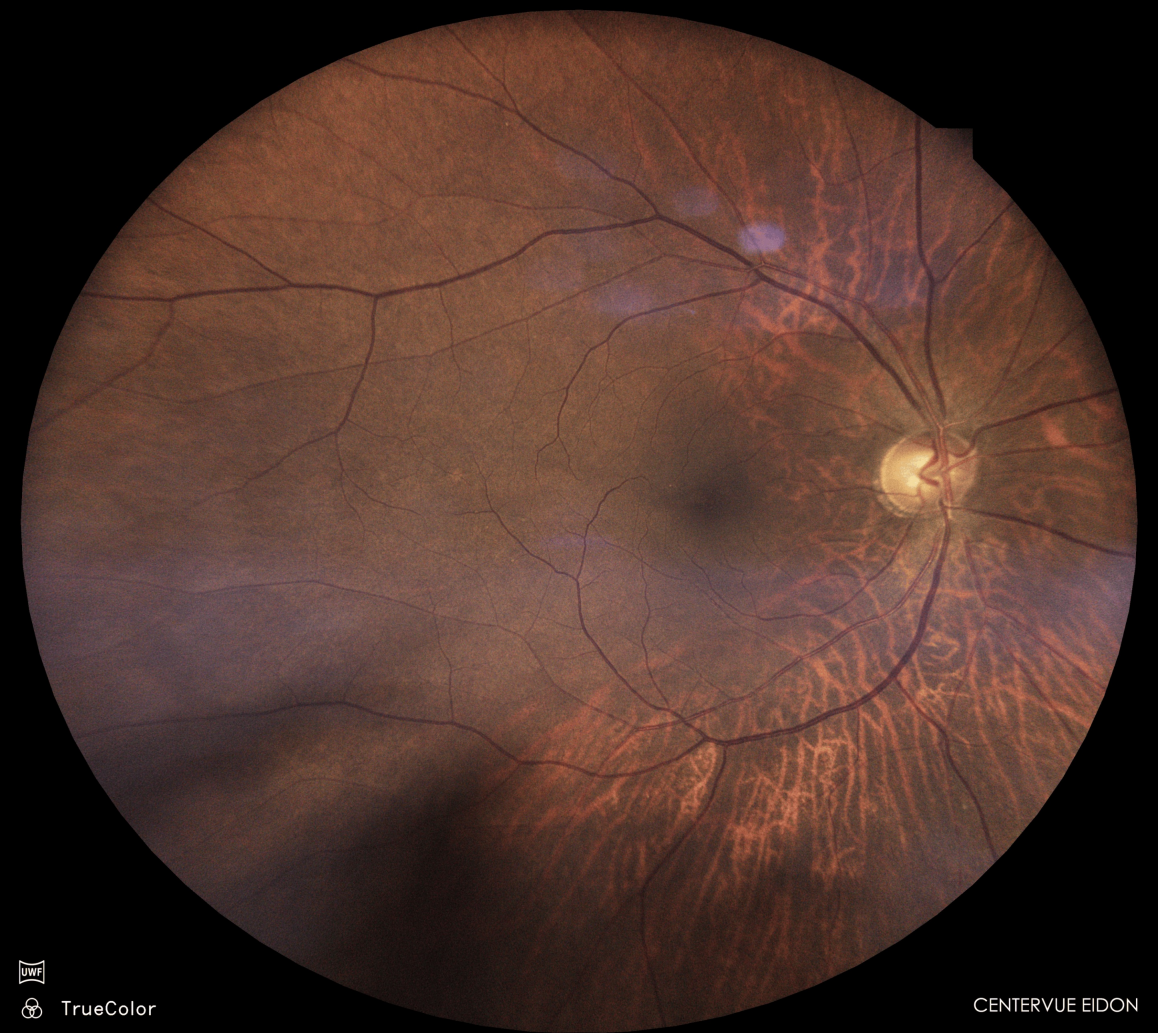
Color fundus photograph of the right eye - clinically unremarkable.

**Figure 2:**
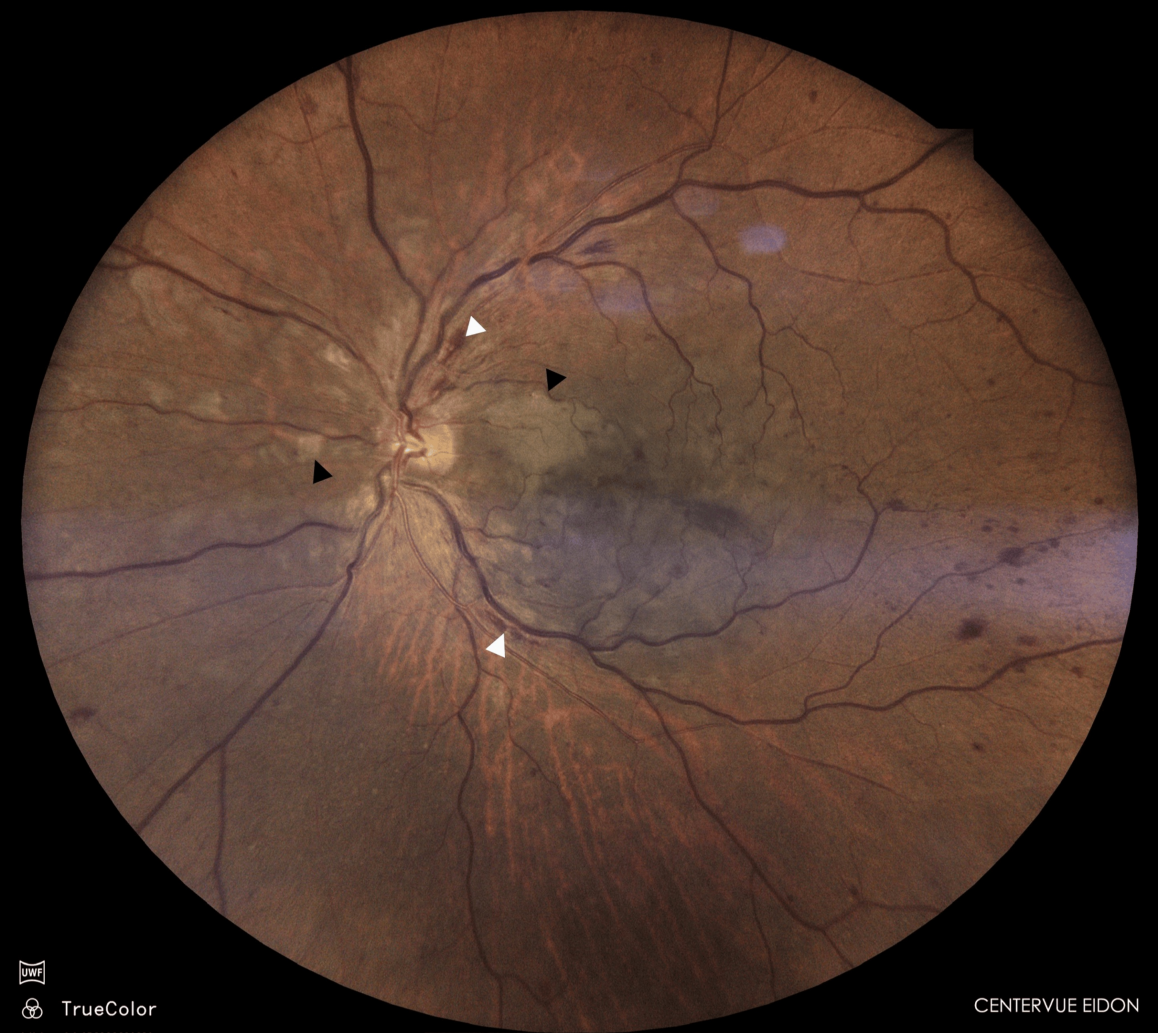
Color fundus photograph of the left eye demonstrating multiple superficial intraretinal hemorrhages (white arrowheads), numerous cotton wool spots, and characteristic Purtscher flecken (black arrowheads).

**Figure 3:**
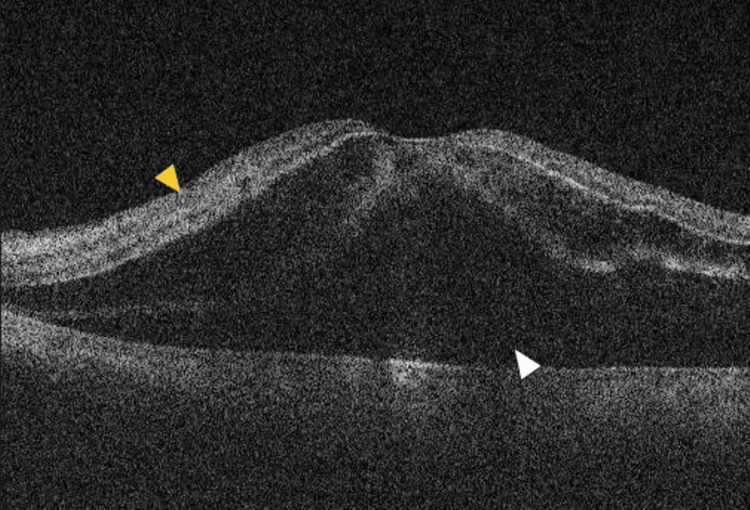
Optical coherence tomography (OCT) of the left macula showing macular edema (white arrowhead) with inner retinal hyperreflectivity (yellow arrowhead) corresponding to the areas of infarction.

On comprehensive ophthalmic examination, best corrected visual acuity (BCVA) was 6/9 in the right eye and 2/60 in the left eye. Anterior segment evaluation was unremarkable bilaterally, showing only early lenticular changes. Dilated fundus examination of the right eye was within normal limits. The left eye revealed multiple superficial intraretinal hemorrhages, numerous cotton wool spots, and characteristic Purtscher flecken (discrete areas of inner retinal whitening with a clear zone surrounding the retinal arterioles) scattered throughout the posterior pole. Clinically significant macular edema accompanied these findings (Figures 1-3).

Systemic evaluation, including a comprehensive metabolic panel, complete blood count, and coagulation profile, was unremarkable at the time of presentation, specifically with a normal platelet count (208 x 10^9^/L), stable hemoglobin (11.0 g/dL), and normal serum creatinine (0.9 mg/dL) with no immediate systemic signs of overt hemolytic uremic syndrome (HUS) or thrombotic thrombocytopenic purpura (TTP).

Based on the classic funduscopic triad of cotton wool spots, retinal hemorrhages, and Purtscher flecken in the setting of recent gemcitabine and bevacizumab administration, a diagnosis of unilateral PLR was established. The clinical picture was differentiated from a combined vascular occlusion by the distinct morphology of the retinal whitening. Unlike central retinal artery occlusion, the patient lacked profound arterial attenuation or box-carring, exhibiting pathognomonic Purtscher flecken with characteristic perivascular clearing instead. Furthermore, the absence of severe venous dilation, tortuosity, and diffuse four-quadrant hemorrhages effectively ruled out central retinal vein occlusion.

Following a multidisciplinary discussion with the patient's oncologist, the condition was attributed to a localized, drug-induced thrombotic microangiopathy, with gemcitabine being the primary suspected inciting agent. Given the self-limiting nature of PLR, conservative management with close clinical follow-up was advised. The patient was scheduled for a review in two weeks to monitor for spontaneous resolution. Her chemotherapeutic regimen was temporarily withheld for one month in consultation with her treating oncologist. Due to the patient having exhausted multiple other chemotherapeutic regimens in previous cycles, the decision was made to eventually restart the same regimen.

**Figure 4:**
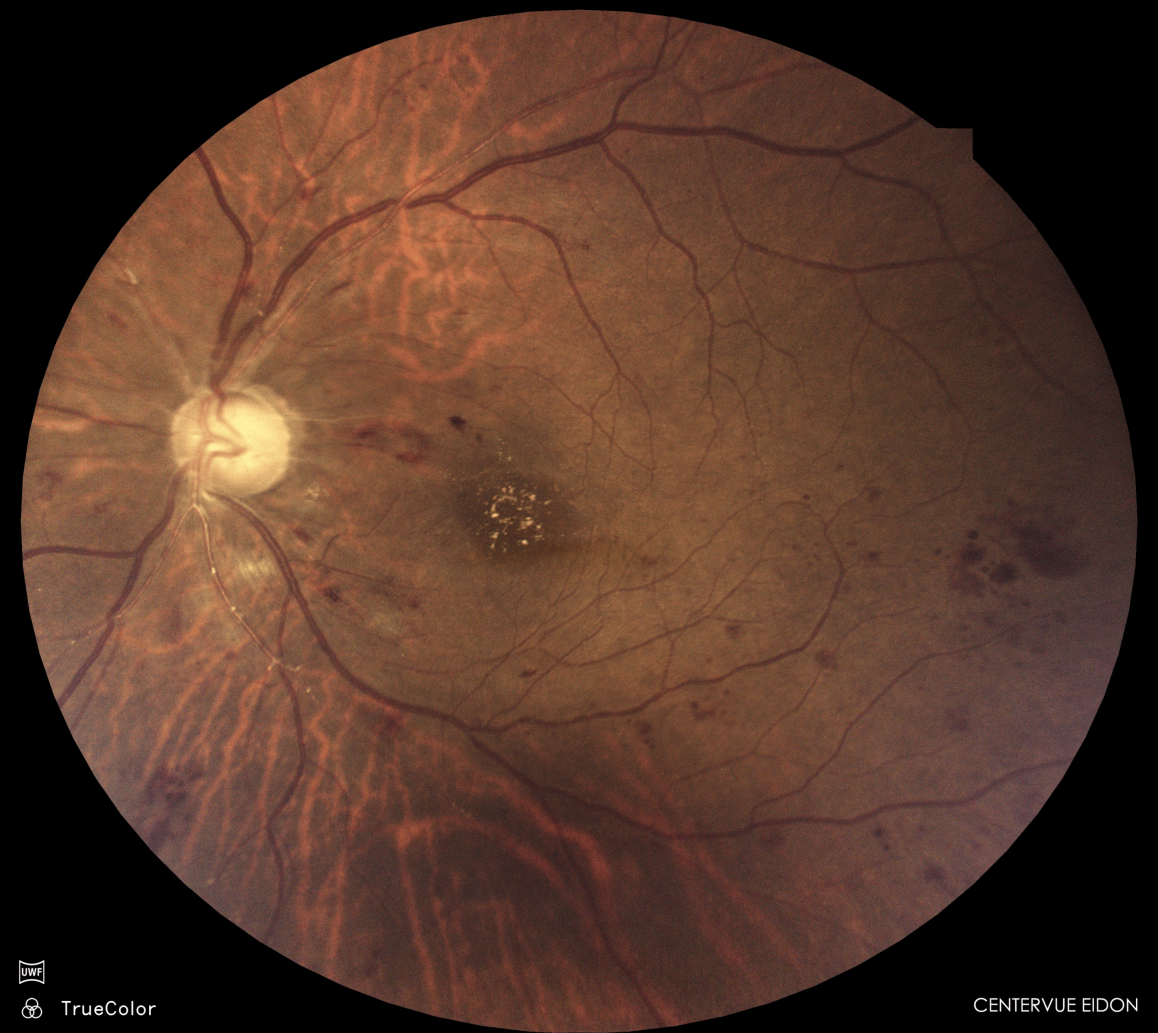
Left eye fundus photograph at two-month follow-up: Resolved Purtscher flecken and macular edema, hard exudates at the fovea.

**Figure 5:**
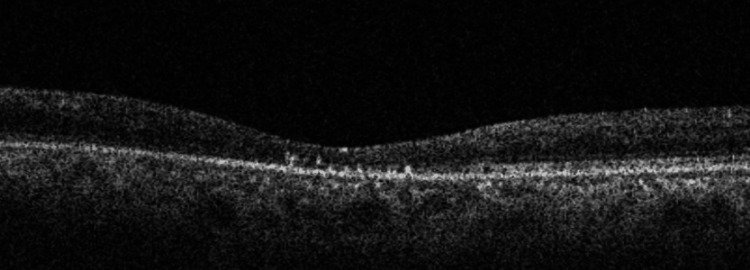
Left eye macular optical coherence tomography (OCT) at two-month follow-up showing foveal thinning and hard exudates.

At the two-month follow-up examination, the patient's visual acuity in the left eye remained unchanged. Repeat OCT of the left macula demonstrated a complete resolution of the macular edema with resultant foveal thinning and hard exudates at the fovea (Figures 4-5).

The right eye remained clinically unremarkable. Given the pathognomonic clinical findings on fundus examination and structural OCT, additional ancillary testing such as fundus fluorescein angiography (FFA) or optical coherence tomography angiography (OCTA) was not deemed strictly necessary for diagnosis.

## Discussion

The pathophysiology of PLR involves complement activation and leukoembolization, leading to the occlusion of precapillary arterioles and subsequent microvascular infarction [6-9]. In the context of oncology, a hypercoagulable paraneoplastic state combined with the endothelial toxicity of chemotherapeutic agents creates a high-risk environment for such microvascular events.

Gemcitabine is a well-established cause of drug-induced TMA [2,6]. The endothelial damage is thought to be immune-mediated or a direct toxic effect, precipitating platelet aggregation and microthrombi formation. Bevacizumab, a systemic anti-VEGF agent, can independently induce TMA by depleting VEGF, which is critical for endothelial cell survival and fenestration maintenance [7]. The synergistic endothelial toxicity of introducing both gemcitabine and bevacizumab likely triggered a localized retinal TMA in this patient.

A critical aspect of this case was the initial misdiagnosis of a combined CRAO/CRVO. While both PLR and combined occlusions present with profound visual loss, retinal hemorrhages, and whitening, careful morphological differentiation is required. Purtscher flecken represent focal, discrete infarctions that classically spare the immediate periarteriolar space, distinguishing them from the diffuse, confluent inner retinal ischemic pallor with a cherry-red spot typical of CRAO. Differentiating these entities is vital, as the systemic implications, investigations, and treatments differ vastly.

The unilateral presentation in this patient is noteworthy, as PLR is frequently bilateral, though unilateral cases are documented [5]. The pathophysiology of PLR involves complement activation, specifically C5a, which induces massive leukocyte aggregation and leukoembolization. The unilateral nature of this case can be attributed to the asymmetric hemodynamic distribution of these microemboli. Similar to embolic phenomena in cholesterol emboli (Hollenhorst plaques) or asymmetric embolic strokes, the leukoemboli in PLR may preferentially shower into the ophthalmic artery circulation of a single eye based on localized rheological factors, carotid hemodynamics, or unilateral variations in precapillary arteriolar caliber, sparing the contralateral eye entirely.

Prior intracavitary brachytherapy and earlier systemic chemotherapy may have altered the baseline vascular integrity, establishing a predilection for the acute event upon exposure to the new drug regimen in this case.

Given the unilateral presentation, it is critical to differentiate PLR from other acute unilateral vascular occlusions, namely, CRAO and CRVO. Clinically, CRVO is characterized by dilated, tortuous retinal veins and extensive, deep retinal hemorrhages spanning all four quadrants; in contrast, our patient exhibited normal venous caliber with pathology strictly localized to the posterior pole. Furthermore, while CRAO presents with diffuse, uniform inner retinal whitening and a classic cherry-red spot, PLR is distinguished by discrete Purtscher flecken - focal areas of inner retinal whitening that characteristically leave a clear, unaffected zone immediately surrounding the retinal arterioles. The presence of these pathognomonic flecken, combined with the absence of gross venous stasis or global retinal infarction on multimodal imaging, firmly established the diagnosis of PLR and ruled out primary central retinal occlusive events.

Management of PLR remains largely observational [5,8] with visual recovery largely dependent on the resolution of the underlying systemic trigger [10-13]. Addressing the underlying systemic trigger - in this case, discussing the chemotherapeutic regimen with the treating oncologist - is paramount. The spontaneous, gradual visual recovery noted in many cases often justifies initial conservative management.

## Conclusions

PLR is a rare but severe ocular complication that can arise following the administration of gemcitabine and bevacizumab. This case underscores the necessity for precise evaluation to differentiate PLR from other catastrophic vascular events such as combined occlusions. Early recognition of drug-induced localized thrombotic microangiopathy allows for prompt multidisciplinary coordination with oncology, ensuring comprehensive patient care.

## References

[1] Agrawal A, McKibbin M (2007). Purtscher's retinopathy: epidemiology, clinical features and outcome. Br J Ophthalmol.

[2] Parc C (2007). Purtscher-like retinopathy as an initial presentation of a thrombotic microangiopathy associated with antineoplastic therapy. Am J Hematol.

[3] Shah RJ, Choudhry N, Leiderman YI (2013). Purtscher-like retinopathy in association with metastatic pancreatic adenocarcinoma and capecitabine therapy. Retin Cases Brief Rep.

[4] Rai M, Shiva Shiva, Gupta S, Gupta SK (2025). Chemotherapy-induced bilateral Purtscher-like retinopathy in metastatic breast carcinoma - a case report and review of literature. Indian J Ophthalmol Case Rep.

[5] Proença Pina J, Ssi-Yan-Kai K, de Monchy I, Charpentier B, Offret H, Labetoulle M (2008). Purtscher-like retinopathy: case report and review of the literature [Article in French]. J Fr Ophtalmol.

[6] Sheyman AT, Wald KJ, Pahk PJ, Freund KB (2014). Gemcitabine associated retinopathy and nephropathy. Retin Cases Brief Rep.

[7] Chiu CY, Cheng CK (2023). Synergic effect of intravitreal bevacizumab and systemic corticosteroid in treating systemic inflammatory response syndrome (SIRS) associated Purtscher-like retinopathy. Ocul Immunol Inflamm.

[8] Espinosa-Barberi G, Alba Linero C, Llorens Bellés V, Adán Civera A (2019). Multimodal imaging and treatment of Purtscher-like retinopathy. Arch Soc Esp Oftalmol (Engl Ed).

[9] Agrawal A, McKibbin MA (2006). Purtscher's and Purtscher-like retinopathies: a review. Surv Ophthalmol.

[10] Miguel AI, Henriques F, Azevedo LF, Loureiro AJ, Maberley DA (2013). Systematic review of Purtscher's and Purtscher-like retinopathies. Eye (Lond).

[11] Serhan HA, Abuawwad MT, Taha MJ (2024). Purtscher's and Purtscher-like retinopathy etiology, features, management, and outcomes: a summative systematic review of 168 cases. PLoS One.

[12] Gahn GM, Khanani AM, Khan M (2020). Purtscher's-like retinopathy associated with acute pancreatitis. Am J Ophthalmol Case Rep.

[13] Bakka HS, Babu PK, Kutikuppala LV, Reddy MV, Varshitha G (2024). Purtscher's like retinopathy - a rare ocular complication of acute pancreatitis. Int J Surg Case Rep.

